# The Impact of Blood Flow Restriction Training on Glucose and Lipid Metabolism in Overweight or Obese Adults: A Systematic Review and Meta-Analysis

**DOI:** 10.3390/life15081245

**Published:** 2025-08-06

**Authors:** Hao Chen, Peng Liu, Yidi Deng, Haibo Cai, Pu Liang, Xin Jiang

**Affiliations:** 1 Physical Education Department, Dalian University of Finance and Economics, Dalian 116622, China; chenhao@dlufe.edu.cn; 2 College of Physical Education, Dalian University, Dalian 116622, China; liupeng@s.dlu.edu.cn (P.L.); dengyidi@s.dlu.edu.cn (Y.D.); caihaibo@s.dlu.edu.cn (H.C.); liangpu@s.dlu.edu.cn (P.L.)

**Keywords:** blood flow restriction therapy, glucolipid metabolism, obesity, adult, meta-analysis

## Abstract

Blood flow restriction training (BFRT) offers notable advantages, including simplicity and time efficiency. However, no meta-analysis has yet comprehensively evaluated its effects on glucose and lipid metabolism in overweight or obese adults. This meta-analysis examines the potential efficacy of BFRT in improving glycemic and lipid control in overweight/obese adults. The literature was searched in six databases, with the search period up to 31 March 2025. A total of eight randomized controlled trials involving 267 participants were identified. Data were analyzed using Stata 18.0 and RevMan 5.4 with random effects models. Outcomes included fasting blood glucose (FBG), homeostasis model assessment of insulin resistance (HOMA-IR), and lipid profiles, and risk of bias and publication bias (Egger’s test) were assessed. BFRT significantly reduced FBG (Hedges’ g = −1.13, 95% CI: −1.65 to −0.62, *p* < 0.01; *I*^2^ = 66.34%) and HOMA-IR (Hedges’ g = −0.98, 95% CI: −1.35 to −0.61, *p* < 0.01; *I*^2^ = 17.33%) compared with the controls. However, no significant changes were observed in lipid profiles. Our analysis demonstrates that BFRT exhibits the favorable effect of improving glucose metabolism in overweight/obese adults; however, current evidence does not support significant advantages of BFRT for lipid metabolism improvement.

## 1. Introduction

Overweight and obesity have emerged as a global health crisis in the 21st century, posing substantial threats to public health worldwide [1]. In 2021, overweight and obesity were attributable to 3.71 million deaths and 129 million globally, accounting for 5.7% of total mortality and 4.9% of global disease burden [2]. Projections indicate that obesity prevalence will surpass 57.8% in over 70 countries by 2030 [3]. Obesity is now internationally recognized as a complex chronic disease [4]. The rising prevalence of obesity has contributed to an increased burden of non-communicable diseases (NCDs), particularly diabetes, cardiovascular diseases (CVDs), and cancer [5]. The estimated economic costs of obesity-related conditions range from USD 319 million in low-income countries to USD 1.33 trillion in high-income nations [6]. These consequences not only diminish the quality of life and elevate health risks but also represent a critical global public health challenge [7]. CVD remains the leading cause of global mortality [5,8,9]. Individuals with obesity exhibit elevated LDL-C oxidation, dyslipidemia, reduced muscle mass, and other cardiovascular risk factors that collectively promote CVD development [10,11]. Patients with obesity show significant associations between reduced muscle strength and elevated triglycerides, total cholesterol, and glucose levels [12]. The pro-atherogenic lipid profile associated with obesity—characterized by elevated triglycerides (TG), increased low-density-lipoprotein cholesterol (LDL-C), and reduced high-density-lipoprotein cholesterol (HDL-C)—plays a significant role in CVD pathogenesis [13]. HDL-C serves as both a biomarker of CVD [14] and as ‘good cholesterol’, offering protective effects on overall health, particularly cardiovascular health [15].

The dramatic increase in obesity prevalence is largely driven by four modifiable risk factors: poor dietary habits, physical inactivity, sedentary behavior, and inadequate sleep duration [16]. Regular physical activity confers cardiometabolic benefits via multiple mechanisms: (1) optimizing blood composition and lipid metabolism [17] and (2) enhancing insulin sensitivity and glucose homeostasis [18], which collectively lead to reduced adiposity and lower CVD risk [19]. Exercise is well-established as an effective non-pharmacological intervention for both the treatment and prevention of various chronic diseases [20]. For example, combining aerobic exercise with dietary modifications produces significant cardiometabolic improvements in individuals with obesity [21,22]. Research has shown that high-intensity interval training (HIIT) significantly improves glucose regulation and lipid metabolism [23]. Aerobic exercise significantly enhances lipid profiles in overweight/obese individuals by favorably altering TC, TG, LDL-C, and HDL-C levels, thereby improving lipid metabolism [21,24]. Furthermore, vigorous physical activity (VPA) performed in 10 min bouts, two–three times per week, improves glucose and lipid metabolism while enhancing life expectancy [25].

From a practical standpoint, individuals with overweight/obesity frequently encounter barriers to vigorous exercise participation, including chronic physical inactivity and limited exercise experience [26]. BFRT involves the application of external pressure to proximal limbs via specialized cuffs or inflatable devices during exercise [27]. This technique promotes muscle hypertrophy and strength gains at low-to-moderate intensities through enhanced BFR-mediated anabolic processes [28]. Research has shown that BFR combined with slow-walk training stimulates muscle hypertrophy and strength improvements in young males [29]. When combined with resistance exercise, BFRT promotes muscle hypertrophy, increases strength [30,31,32], and reduces exercise-induced muscle damage [33]. BFRT paired with aerobic exercise significantly increases maximal oxygen uptake (VO_2max_) and improves muscular strength [34]. BFRT shows clinically significant rehabilitative effects for knee joint recovery [35]. Furthermore, BFRT confers cardiovascular benefits, including reduced blood pressure [36], improved body composition (e.g., body fat percentage and BMI), and enhanced lipid metabolism (FBG, TC, TG, LDL-C, and HDL-C) [37]. BFRT supports fat reduction and weight management in individuals with overweight/obesity by improving body composition, optimizing lipid metabolism, enhancing physical fitness and muscle performance, and ultimately lowering injury risk [37]. During BFRT interventions, participants typically exhibit reduced mechanical loading, faster recovery [38], and minimal exercise-induced muscle damage [39].

To date, no comprehensive studies have systematically examined the effects of BFRT on glucolipid metabolism in adults with overweight or obesity. Consequently, this study conducts a systematic review and meta-analysis to determine whether BFRT provides superior improvements in glucolipid metabolic parameters relative to both non-exercise controls and conventional training programs. This systematic review and meta-analysis evaluated the effects of BFRT on glucolipid metabolism in overweight or obese adults by comparing BFRT with conventional training and non-exercise controls. Primary outcomes included FBG and HOMA-IR; secondary outcomes covered lipid profiles (TC, TG, HDL-C, and LDL-C).

## 2. Materials and Methods

### 2.1. Design

This systematic review was conducted in strict accordance with the Preferred Reporting Items for Systematic Reviews and Meta-Analyses (PRISMA) guidelines [40]. The study protocol was prospectively registered in PROSPERO (Registration ID: CRD420251026882).

### 2.2. Literature Search

The eligible studies were selected independently by two authors (H.C. and P.L.). Disagreements were resolved by a third author (X.J.). A systematic search was performed in the databases: CNKI, PubMed, Web of Science, Embase, Scopus, and Cochrane Library, covering all available records from their inception to 31 March 2025. Relevant search terms were combined using Boolean operators (AND, OR). The detailed search strategies and results are provided in Table S1. Additionally, we manually screened the reference lists of all included studies and meta-analyses to identify potentially eligible studies that might have been missed in the database searches.

### 2.3. Literature Selection

All identified records retrieved from the database searches were imported into EndNote X9 for automated duplicate removal, followed by manual verification. Two independent reviewers (H.C. and P.L.) performed a blinded screening of titles and abstracts based on predefined eligibility criteria to exclude irrelevant studies. Articles deemed potentially eligible underwent full-text assessment, during which both reviewers independently evaluated each study against the PICOS (Population, Intervention, Comparison, Outcomes, and Study design) framework. Discrepancies between reviewers were resolved through consensus or by consultation with a third author (X.J.) when necessary. To mitigate potential selection bias, no language restrictions were applied; non-English articles were professionally translated prior to evaluation.

### 2.4. Inclusion and Exclusion Criteria

Inclusion criteria were established according to the PICOS framework: (1) Participants: Adults (≥18 years) with overweight or obesity, free from mobility limitations. (2) Intervention: The experimental group underwent BFRT, while control groups received either general training (GT) or blank controls (BCs); minimum intervention duration: 2 weeks. (3) Methodology: BFRT implementation requiring specialized occlusion cuffs/bands applied to proximal upper or lower extremities. (4) Study design: Published randomized controlled trials (RCTs) with clinical outcomes. (5) Primary metabolic outcomes including TC, TG, HDL-C, LDL-C, FBG, and HOMA-IR.

Exclusion Criteria: (1) Research that does not correspond to the topic; (2) reviews, systematic reviews, and meta-analyses of the literature; (3) studies lacking reported data on our predefined metabolic outcomes; (4) non-overweight or obese adults and use of any medications within 6 months before enrollment, with inconsistent dietary patterns between pre- and post-experimental phases; (5) single-arm experiments and subjects that participated in any activities outside the prescribed intervention protocol.

### 2.5. Literature Screening and Data Extraction

Two authors (H.C. and P.L.) systematically extracted the following parameters for all outcome measures: (1) pre-intervention data: mean (M), standard deviation (SD), and sample size (n); (2) post-intervention data: mean (M), standard deviation (SD), and sample size (n). In studies where standard errors (SEMs) were reported, the standard deviation (SD) was calculated using the equation SD = SEM × $\sqrt{N}$, where SD represents the standard deviation, SEM denotes the standard error of the mean, and N refers to the sample size. Subsequently, the standard deviation of the mean difference was calculated as follows:

$$SD_{\mathrm{diff}}=\sqrt{S{D^{2}}_{\mathrm{pre}}+S{D^{2}}_{\mathrm{post}}-2r\times SD_{\mathrm{pre}}\times SD_{\mathrm{post}}}$$

Here, SD_diff_ represents the standard deviation of the differences between pre–post-intervention values. SD_pre_ and SD_post_ stand for the standard deviations of the pre-intervention and post-intervention measures, respectively, while r denotes the correlation coefficient between the pre- and post-intervention measurements. When the correlation was not reported, the within-participant correlation coefficient r was defaulted to 0.5.

Values presented as figures were digitized using graph digitizer software (WebPlotDigitizer 4.8), and the means and SDs were measured manually at the pixel level to the scale provided on the figure [41]. Two authors (H.C. and P.L.) extracted the data using a specific sheet, and any disagreements were resolved through discussion with a third author (X.J.).

### 2.6. Risk of Bias Assessment for Included Studies

The Cochrane Collaboration’s RCT bias assessment tool was used to evaluate the following: (1) random sequence generation; (2) allocation concealment; (3) the blinding of participants and personnel; (4) the blinding of outcome assessment; (5) incomplete outcome data; (6) selective reporting; (7) other potential bias sources. The assessment of the risk of bias was carried out independently by two authors (H.C. and P.L.), and any discrepancies were resolved through discussion.

### 2.7. Certainty of Evidence

The Grading of Recommendations Assessment, Development, and Evaluation (GRADE) method was employed to assess the quality of evidence [42]. The GRADE assesses the certainty of evidence to fall into the categories of very low, low, moderate, or high.

### 2.8. Statistical Analysis

Risk bias assessment was performed using RevMan 5.4 software to generate a risk-of-bias graph for the included studies. Meta-analysis and forest plots for outcome measures were conducted using Stata 18 and GraphPad Prism 8 software. All outcome data were continuous variables. Hedges’ g was selected as the effect size measure, with exact computation for bias correction factors and Hedges and Olkin’s method for standard error correction. Effect sizes were interpreted as follows: negligible (<0.2), small (0.2–0.5), medium (0.5–0.8), and large (>0.8). The DerSimonian–Laird random effects model was applied, with a *p*-value < 0.05 considered statistically significant. If substantial heterogeneity *(I*^2^ > 50%) was detected, a leave-one-out sensitivity analysis was performed to assess robustness. Subgroup analysis was conducted based on exercise type. Publication bias was evaluated using Egger’s regression test; a *p*-value < 0.05 indicated significant publication bias, whereas a *p* ≥ 0.05 suggested no evident bias.

## 3. Results

### 3.1. Study Selection

**Figure 1:**
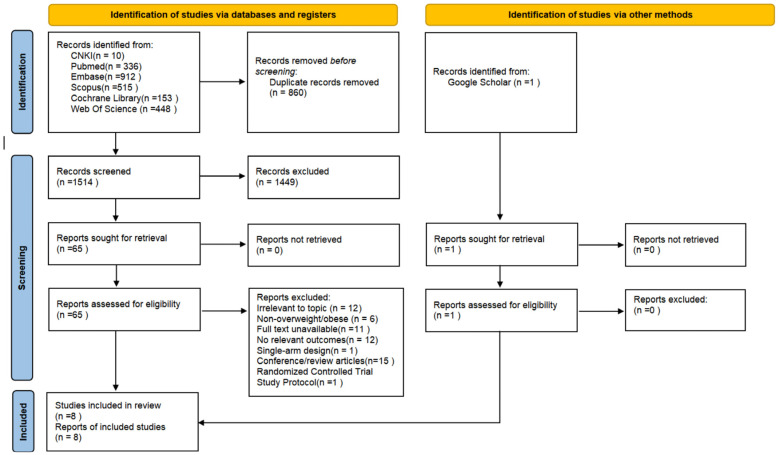
PRISMA flowchart of study selection.

A total of 2375 records were retrieved from the databases. After removing 860 duplicate studies and excluding 1449 irrelevant studies based on title/abstract screening, 65 full-text articles were assessed for eligibility. Ultimately, eight studies met the inclusion criteria and were included in the final meta-analysis (Figure 1). The baseline characteristics of the included studies are summarized in Table 1.

### 3.2. Characteristics of Included Studies

This meta-analysis included 267 overweight or obese adults (178 males and 89 females). The study populations comprised male-only (*n* = 5), female-only (*n* = 2), and mixed-sex (*n* = 1) groups. The studies focused on distinct metabolic phenotypes: obesity (*n* = 2), overweight (*n* = 4), and normal-weight obesity (*n* = 2). Six studies investigated resistance training (RT), while two examined aerobic training (AT). Lipid profiles were assessed as follows: TG, HDL-C, and LDL-C in six studies and TC in five studies. Glucose metabolism parameters included FBG in five studies and HOMA-IR in three studies.

### 3.3. Risk of Bias Assessment Results

**Figure 2:**
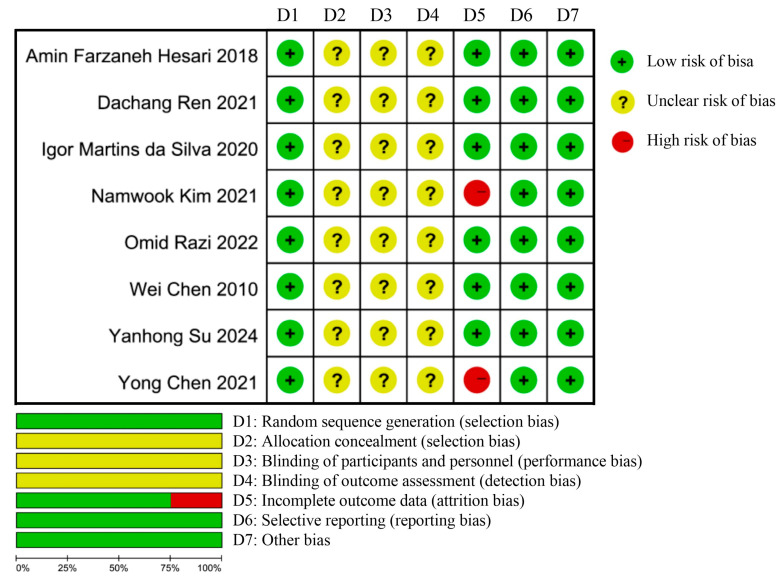
The risk assessment of bias [37,43,44,45,46,47,48,49].

Figure 2 presents the risk of bias assessment for the included studies. All included studies were RCTs. None of the studies reported specific methods for allocation concealment. Given the nature of exercise interventions, all participants provided written informed consent and were advised of pertinent safety precautions. Blinding was not feasible, resulting in an ‘unclear risk’ designation for all studies. Participant withdrawal occurred in two studies during the intervention period.

### 3.4. Meta-Analysis, Certainty of Evidence, and Publication Bias Detection

#### 3.4.1. Glucose Metabolism

**Figure 3:**
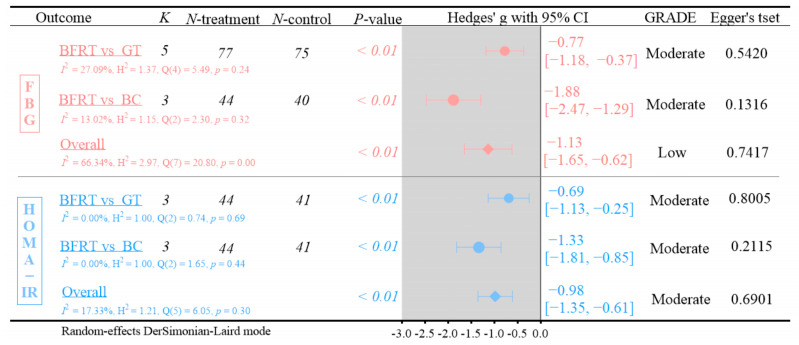
Analysis of outcome measures for FBG and HOMA-IR [37,43,45,47,49].

FBG and HOMA-IR outcomes are shown in Figure 3.

(1) Five RCTs were included in the FBG analysis. Compared with GT, BFRT showed significantly greater reductions in FBG (Hedges’ g = −0.77, 95% CI: −1.18 to −0.37; *p* < 0.01, *I*^2^ = 27.09%; GRADE: moderate; Egger’s test: *p* = 0.5420). Compared with BC, BFR training also demonstrated significant FBG reductions (Hedges’ g = −1.88, 95% CI: −2.47 to −1.29; *p* < 0.01, *I*^2^ = 13.02%; GRADE: moderate; Egger’s test: *p* = 0.1316).
(2) Three RCTs were included in the HOMA-IR analysis. Compared with conventional training, BFRT significantly improved HOMA-IR (Hedges’ g = −0.69, 95% CI: −1.13 to −0.25; *p* < 0.01, *I*^2^ = 0%; GRADE: moderate; Egger’s test: *p* = 0.8005). Compared with BC, BFRT showed greater improvements in HOMA−IR (Hedges’ g = −1.33, 95% CI: −1.81 to −0.85; *p* < 0.01, *I*^2^ = 0.00%; GRADE: moderate; Egger’s test: *p* = 0.2115).

#### 3.4.2. Lipid Metabolism

**Figure 4:**
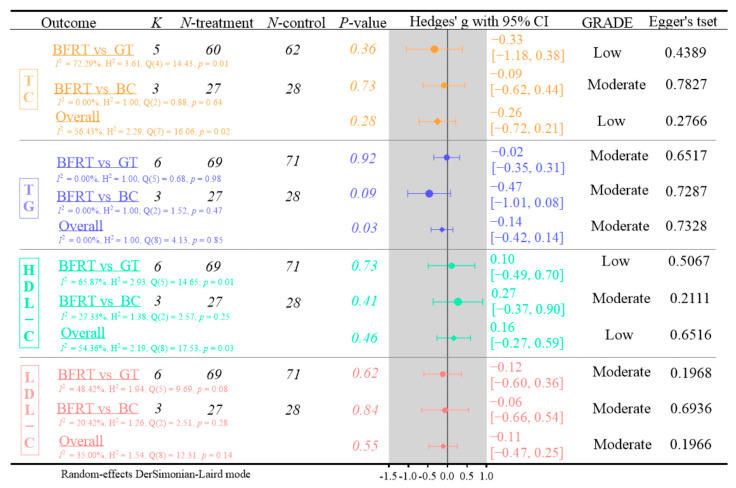
Analysis of lipid profile outcomes (TC, TG, HDL-C, and LDL-C) [37,44,45,46,48,49].

Figure 4 shows the analysis of outcome measures for TC, TG, HDL-C, and LDL-C.

(1) Five RCTs were included in the TC analysis. BFRT demonstrated no significant effects on TC levels compared to GT or BC (Hedges’ g = −0.26, 95% CI: −0.72 to 0.21; *p* = 0.28, *I*^2^ = 56.43%; GRADE: low; Egger’s test: *p* = 0.2766).
(2) Six RCTs were included in the TG analysis. Blood flow restriction (BFR) training showed no significant effects on TG levels compared to GT or BC (Hedges’ g = −0.14, 95% CI: −0.42 to 0.14; *p* = 0.03, *I*^2^ = 0.00%; GRADE: moderate; Egger’s test: *p* = 0.7328).
(3) Six RCTs examined HDL-C levels. This meta-analysis demonstrated no significant effects of BFRT on HDL-C compared to GT or BC (Hedges’ g = 0.16, 95% CI: −0.27 to 0.59; *p* = 0.46, *I*^2^ = 54.36%; GRADE: low; Egger’s test: *p* = 0.6516).
(4) Six RCTs assessed LDL-C levels. This meta-analysis indicated no significant effects of BFRT on LDL-C concentrations compared to GT or no BC (Hedges’ g = −0.11, 95% CI: −0.47 to 0.25; *p* = 0.55, *I*^2^ = 35%; GRADE: moderate; Egger’s test: *p* = 0.1966).

### 3.5. Sensitivity Analysis

A leave-one-out sensitivity analysis was conducted on the outcomes exhibiting substantial heterogeneity (TC and HDL-C), detailed results are shown in Table 2. Specifically, the leave-one-out analysis results revealed the following estimates: TC (Hedges’ g = 0.02, 95% CI −0.40 to 0.45; *p* = 0.92, *I*^2^ = 0%) and HDL-C (Hedges’ g = −0.14, 95% CI −0.61 to 0.33; *p* = 0.57, *I*^2^ = 29%).

### 3.6. Subgroup Analysis

Subgroup analyses were performed based on AT and RT to assess their effects on blood glucose levels. Due to the limited data on AT (only one study), its effect on blood glucose was assessed descriptively. The findings showed that BFRT combined with AT significantly improved blood glucose levels [37]. For RT, the meta-analysis demonstrated that BFRT combined with RT significantly reduced FBG levels (Hedges’ g = −0.64, 95% CI: −1.08 to −0.21; *p* < 0.01, *I*^2^ = 17.51%) [43,45,47,49].

## 4. Discussion

To our knowledge, this is the first systematic review and meta-analysis to comprehensively evaluate the effects of BFRT on glucose and lipid metabolism in adults. The primary objective of this study was to systematically assess whether BFRT provides superior metabolic benefits compared with either non-exercise controls or conventional training regimens in overweight/obese adults. The meta-analysis demonstrated that BFRT significantly improved FBG and HOMA-IR compared with conventional training or no-intervention controls. However, no statistically significant effects were observed for TG, TC, HDL-C, or LDL-C. Subgroup analyses further revealed that BFRT combined with RT yielded significantly greater improvements in FBG and HOMA-IR than RT alone.

### 4.1. Effects on Glucose Metabolism

This meta-analysis revealed that BFRT significantly improved markers of glucose metabolism in overweight/obese adults compared with both GT and BC: FBG (Hedges’ g = −1.13, 95% CI −1.65 to −0.62, *p* = 0.00, *I*^2^ = 66.34%); HOMA-IR (Hedges’ g = −0.98, 95% CI −1.35 to −0.61, *p* < 0.01, *I*^2^ = 17.33%). Both FBG and HOMA-IR demonstrated large effect sizes (>0.8). However, the GRADE assessment revealed differing evidence certainty: low certainty for FBG (downgraded due to risk of bias and imprecision), indicating limited confidence in the estimate, but moderate certainty for HOMA-IR, suggesting that the findings are likely to reflect the true effect. These results corroborate previous evidence indicating that BFRT enhances FBG and HOMA-IR in overweight/obese individuals [49].

Hyperglycemia not only mediates cellular damage but also disrupts lipid synthesis and degradation, thereby exacerbating insulin resistance and perpetuating a vicious cycle of glucose and lipid metabolism [50]. Precise regulation of blood glucose is critical to maintaining health and preventing chronic diseases [51]. BFR intervention exerts positive regulatory effects on glycemic control [52]. When combined with RT, it significantly improves glycemic control in obese or overweight adults [37,49], which aligns with the findings of the present study. HIIT, a time-efficient exercise modality, has been shown to improve glycemic control after a training period, yielding benefits comparable to moderate-intensity continuous training [53]. However, resistance exercise induces more pronounced reductions in blood glucose concentrations immediately post-exercise and 30 min post-exercise compared with HIIT [51]. Elevated body mass in overweight/obese populations often reduces compliance with aerobic exercise, whereas resistance-based training demonstrates higher acceptability [54]. Both aerobic and resistance exercise improve glycemic control [55], though aerobic exercise-induced energy expenditure may contribute to transient changes in glucose and insulin levels, with both parameters exhibiting a negative correlation [56]. Notably, significant reductions in blood glucose were observed after 45 min of resistance exercise compared with the resting state [57]. Cyclic resistance exercise protocols elevate circulating METRNL concentrations at rest [58], and this increase in serum METRNL levels correlates significantly with reductions in FBG [59]. Subgroup analysis in this study revealed that BFR combined with RT produced superior FBG improvements in obese/overweight adults compared with other interventions. Previous studies report minimal blood glucose alterations following aerobic exercise [60], suggesting its limited efficacy in improving FBG levels. Thus, resistance exercise is recommended as a more effective intervention for glycemic control, consistent with the present subgroup analysis. Low-to-moderate intensity RT appears optimal for improving FBG levels [61].

Existing studies indicate that BFRT effectively improves glucose metabolism by enhancing insulin sensitivity, thereby reducing the risk of CVD events [49]. HOMA-IR is primarily determined by the interaction between FBG and fasting insulin (FINS) concentrations [59]. Combining BFR with RT significantly improves HOMA-IR values compared with conventional training [49,62], which aligns with the findings of this study. Previous research has shown that RT effectively reduces resting insulin levels and enhances insulin sensitivity [63]. Cyclic RT significantly increases resting serum METRNL levels, which correlate with reduced HOMA-IR values [58]. Higher-load RT produces similar effects in individuals with type 2 diabetes (T2D) [64,65], potentially due to upregulated glucose transporter (GLUT) proteins and increased insulin receptor density in muscle cells [66]. However, the reduction in HOMA-IR may be time-dependent, as one study reported no significant changes after 12 weeks of RT [67]. In contrast, this study observed significant improvements, possibly due to longer intervention durations in the included literature. While aerobic exercise enhances HOMA-IR and improves FBG [59], this effect was not observed here, likely because all eligible studies exclusively examined RT, precluding sensitivity analysis for HOMA-IR. The present study confirms that BFRT significantly benefits both FBG and HOMA-IR. Based on current evidence, we recommend combining BFRT with RT to optimize glucose metabolism. The present study revealed that the large effect sizes for FBG (Hedges’ g = −1.13) and HOMA-IR (Hedges’ g = −0.98) exceeded the conventional threshold for clinical significance. These findings suggest that the intervention may effectively reduce fasting blood glucose and insulin resistance, which could have beneficial implications for clinical research.

### 4.2. Effects on Lipid Metabolism

No statistically significant differences were observed in any lipid parameters (TC, TG, HDL-C, or LDL-C) when comparing BFRT with both BC and GT groups. The meta-analysis revealed a small but statistically non-significant reduction in TC levels (Hedges’ g = −0.26, 95% CI: −0.72 to 0.21; *p* = 0.28). According to the GRADE framework, the evidence was rated as low certainty, reflecting limited confidence in the effect estimate due to the potential risk of bias. Similarly, the effect on TG levels was negligible and non-significant (Hedges’ g =−0.14, 95% CI: −0.42 to 0.14; *p* = 0.33), with moderate-certainty evidence suggesting moderately reliable findings. For HDL-C levels, the analysis indicated a trivial, non-significant increase (Hedges’ g = 0.16, 95% CI: −0.27 to 0.59; *p* = 0.46), supported by low-certainty evidence. Finally, LHedges’ gL-C levels exhibited a small, non-significant reduction (Hedges’ g = −0.11, 95% CI: −0.47 to 0.25; *p* = 0.55), with moderate-certainty evidence suggesting that these findings likely approximate the true effect. Regular exercise participation beneficially modifies specific components of the lipid profile [64,68]. Exercise reduces visceral adipose tissue stored around abdominal organs, contributing to decreased TG and LDL-C levels [60], thereby improving blood lipid profiles [24]. Furthermore, exercise increases LDL-C particle size and density, reducing their atherogenic potential [69]. The underlying mechanisms for these lipid profile improvements may involve upregulated peroxisome proliferator-activated receptor gamma (PPARγ) and peroxisome proliferator-activated receptor gamma coactivator 1-alpha (PGC-1α) mRNA expression in muscle and adipose tissue following exercise training [67]. Studies indicate that combined functional training (CFT) significantly improves blood lipid profiles [70]. Notably, the combination of moderate-intensity aerobic exercise with low-to-moderate-load RT produces the most substantial improvements in TC and LDL-C levels [61].

Our findings indicate that BFRT provides no additional benefits for lipid metabolism in overweight or obese adults when compared to blank control or routine training conditions, consistent with previous research [44]. Prior investigations have similarly demonstrated no significant differences in TG levels following aerobic exercise conducted under either hypoxic or normoxic conditions [18]. Comparative analyses of AT, RT, and combined training modalities have likewise revealed no significant effects on blood lipid profiles [55,71]. More substantial modifications to blood lipid profiles may require either higher exercise volumes or greater training intensity [72]. Notably, existing evidence suggests that exercise training volume exerts a more pronounced effect than exercise intensity on blood lipid profile improvements [73].

Aerobic exercise induces only modest modifications in TG and LDL-C levels, whereas combined exercise yields slight improvements in HDL-C [60]. Current evidence suggests that significant reductions in LDL-C levels during aerobic exercise interventions occur exclusively when accompanied by body weight loss [74]. Aerobic exercise enhances insulin sensitivity, thereby improving blood glucose regulation while simultaneously reducing hepatic TG and very-low-density-lipoprotein (VLDL) production, ultimately promoting favorable lipid profile modifications [59]. During physical activity, aerobic exercise preferentially utilizes fat as its primary energy substrate [60]. The mechanistic basis for these lipid profile improvements likely involves enhanced fat oxidation mediated by increased exercise intensity and duration, which reduces overall adiposity and optimizes blood lipid composition [75,76]. Elevated HDL-C may reduce blood glucose levels through multiple pathways, principally via activation of the AMP-activated protein kinase (AMPK) signaling pathway and enhanced pancreatic β-cell insulin secretion, consequently promoting skeletal muscle glucose uptake [77]. Furthermore, dyslipidemia may compromise pancreatic functions and those of other organs by impairing both insulin secretion and peripheral insulin sensitivity [51].

Emerging evidence suggests that RT may demonstrate superior efficacy to AT in modulating blood lipid profiles [66]. RT interventions elicit substantial improvements in muscular strength and endurance, typically ranging from 25% to 100% or more [78]. Notably, RT appears particularly effective in elevating HDL-C levels [70]. The therapeutic benefits of RT extend to enhanced muscular strength, endurance capacity, cardiovascular function, and appetite regulation in obese populations [13]. Crucially, RT-mediated maintenance of lean muscle mass enhances basal metabolic rate and preserves insulin homeostasis independently of weight reduction, thereby attenuating hepatic TG accumulation [79]. The accretion of lean muscle mass following RT assumes critical importance for augmenting insulin sensitivity, optimizing fatty acid metabolism, and elevating resting metabolic rate (RMR) [73]. Clinically meaningful improvements in blood lipid profiles are observed when obese individuals achieve significant increases in lean body mass [80]. While meta-analytic data confirm that RT significantly reduces LDL-C concentrations in adult populations [81,82], our findings align with alternative evidence indicating no significant alteration in LDL-C levels following RT [60]. Training interventions that are shorter than 12 weeks generally fail to modify blood lipid and lipoprotein parameters [83], likely due to insufficient stimulus duration to induce meaningful physiological adaptations [84].

### 4.3. Future Research Directions

While this study demonstrated that BFRT significantly improved FBG and HOMA-IR in overweight/obese individuals, the metabolic benefits were comparable regardless of whether BFR was combined with aerobic or resistance exercise. The specific effects of BFR on lipid metabolism parameters remain inconclusive and warrant further investigation. Future investigations should employ randomized controlled trials to systematically evaluate the differential effects of various BFR-combined exercise modalities (e.g., resistance vs. AT) on blood lipid metabolism. Additionally, longitudinal studies with larger sample sizes (n ≥ 30 per group) and extended intervention periods (≥15 weeks) would improve the statistical power and clinical relevance of the findings. Such investigations would facilitate the development of optimized BFR exercise prescriptions for mitigating dyslipidemia risk in overweight and obese populations. Additionally, subsequent studies should aim to establish evidence-based training protocols that maximize improvements in glucolipid metabolism for this population.

### 4.4. Limitations

This study has several limitations. First, the small number of eligible studies (*n* = 8) and limited sample size (*N* = 267) may reduce statistical power. Second, participant attrition in two trials could affect robustness and generalizability. Methodological limitations included a high risk of performance bias (due to unblinded participants) and a lack of allocation concealment in several studies, potentially impacting validity. The evidence on glycemic outcomes was particularly limited—only one study assessed AT, while others focused on RT. The small number of studies precluded subgroup analyses or assessment of optimal training parameters (e.g., intensity and duration). Although findings support BFR combined with RT for improving FBG and HOMA-IR, conclusions remain tentative due to methodological constraints and limited evidence. Finally, the imputation of r = 0.5 for missing correlations is conventional; we acknowledge that this assumption could influence pooled effect sizes if actual correlations are heterogeneous.

## 5. Conclusions

Compared with GT and BC, BFRT demonstrates the favorable effect of improving glucose metabolism in overweight or obese adults, and the combination of BFRT with RT may represent an optimal intervention strategy. However, BFRT does not exhibit a potential advantage in improving lipid metabolism. Due to limitations in the quantity and quality of included studies, these findings require further validation through higher-quality research.

## Figures and Tables

**Table 1:** Main characteristics of studies included in the meta-analysis.

Study	Participant Characteristics				BFR Details		Protocols						Outcome
	Age (M ± SD)/year	BMI (M ± SD)/kg/m^2^	M/F	N	Position	Pressure	Intervention	Intensity	Sets × Reps or Span	Interval	Frequency	Duration (weeks)	
1 Chen (2010a) [43]	BFR: 21.9 ± 2.07 GT: 21.6 ± 1.71	29.45 ± 1.61 29.57 ± 1.42	13/0	7	PT, PUL	120 mmHg	LI-RT	20% 1 RM	4 × 12	2 min	3 t/wk	18	FBG, HOMA-IR
1 Chen (2010b) [43]	BFR: 21.9 ± 2.07 BC: 21.1 ± 1.66	29.45 ± 1.61 29.83 ± 1.83	13/0	7									
2 Farzaneh Hesari et al. (2018a) [44]	18–24	BFR: 27.8 ± 3 GT: 26.4 ± 4	0/18	9	PT, PUL	<70% AOP	LI-RT	30% 1 RM	1 × 30 + 3 × 15	1–4 min	3 t/wk	8	TG, TC, HDL-C, LDL-C
2 Farzaneh Hesari et al. (2018b) [44]		BFR: 27.8 ± 3 BC: 26.7 ± 2	0/19	9									
3 da Silva IM et al. (2020) [45]	BFR: 25.52 ± 2.19 GT: 24.46 ± 2.56	28.45 ± 2.35 28.68 ± 1.76	15/0 15/0	15	PT, PUL	20–40 mmHg	LI-RT	30% 1 RM	4 × 23	2 min	3 t/wk	8	TG, TC, HDL-C, LDL-C, FBG
4 Razi O et al. (2022) [46]	BFR: 44.77 ± 5.09 GT: 42.77 ± 6.35	27.63 ± 1.2 28.36 ± 1.37	9/0 9/0	9	PT, PUL	140–200 mmHg	LI-AT	50 m/min	5 × 2 min	1 min	3 t/wk	8	TG, HDL-C, LDL-C
5 Chen Y et al. (2021) [37]	BFR: 20.3 ± 1.07 GT: 20.1 ± 1.08	30.1 ± 0.95 30.3 ± 1.08	18/0 19/0	18	PT	160–200 mmHg	LI-AT	40% VO_2max_	3 × 15 min	1 min	2 t/wk	12	FBG, TC, TG, HDL-C, LDL-C
6 Ren (2021a) [47]	BFR: 23.77 ± 2.14 CON: 23.73 ± 2.17	30.88 ± 0.26 30.75 ± 0.23	17/11 16/10	28	PT	120–220 mmHg	LI-RT	20–30% 1 RM	10 × (3–4)	2 min	2 t/wk	12	FBG, HOMA-1 R
6 Ren (2021b) [47]	BFR: 23.77 ± 2.14 CON: 23.75 ± 2.16	30.88 ± 0.26 30.89 ± 0.24	17/11 15/11	28	PT								
7 Kim Namwook et al. (2021a) [48]	BFR: 22.3 ± 1.0 GT: 21.5 ± 0.8	met the criteria forormal weight obesity (NWO)	0/9 0/10	9	PT, PUL	n/a	LI-RT	40%1 RM	3 × (14–18)	n/a	2 t/wk	5	TC, TG, HDL-C, LDL-C
7 Kim Namwook et al. (2021b) [48]	BFR: 22.3 ± 1.0 BC: 21.9 ± 0.5		0/9 0/10	9									
8 Su Y et al. (2023a) [49]	20–24	met the criteria forormal weight obesity (NWO)	BFR: 9 GT: 9	9	PT	140–200 mmHg	LI-RT	30%1 RM	4 × (15–30)	30–60 s	5 t/wk	12	FBG, HOMA-IR TC, TG, HDL-C, LDL-C
8 Su Y (2023b) [49]			BFR: 9 BC: 8										

BFR: blood flow restriction group, BC: blank control group, GT: conventional training group, N: sample size of the BFR, PT: proximal thigh, PUL: proximal upper limb, AOP: arterial occlusion pressure, t: times, wk: week, ±: mean and SD, n/a: not available.

**Table 2:** Results of the sensitivity analysis.

Outcome	Eliminate	N	Hedges’ g (95% CI)	*I* ^2^	*p*
TC	Chen (2021) [37]	4	0.02, −0.40 to 0.45	0%	0.92
HDL-C	Chen (2021) [37]	5	−0.14, −0.61 to 0.33	29%	0.57

## Data Availability

All data generated or analyzed during this study are included in the article/Supplementary Materials Section.

## Supplementary Materials

The following supporting information can be downloaded at: https://www.mdpi.com/article/10.3390/life15081245/s1, Table S1: Search strategy.
