# Supplementary material for: The Impact of Blood Flow Restriction Training on Glucose and Lipid Metabolism in Overweight or Obese Adults: A Systematic Review and Meta-Analysis
Source: Life (Basel). 2025 Aug 6;15(8):1245. doi: 10.3390/life15081245 (PMC12387266; doi:10.3390/life15081245)
Supplement: Supplementary file 1 [file life-15-01245-s001.zip › Supplemental materia.doc]

**Supplemental material**

**The Impact of Blood Flow Restriction Training on Glucose and Lipid Metabolism in Overweight or Obese Adults: A Systematic Review and Meta-Analysis**

Table S1. Search strategy	2

Table S1. Search strategy

| Databases | Search strategy | Results |
| --- | --- | --- |
| CNKI | Blood flow restriction + Pressurized training + Blood flow restriction movement +Blood flow restriction  overweight + obesity +Body mass | 10 |
| Pumbed | ((((((((BFRT) OR (BFR)) ) OR (blood flow restriction therapy)) OR (blood flow restriction)) OR (blood flow restriction training)) OR (blood flow restriction exercise)) OR (KAATSU training)) AND (((Overweight) OR (obese)) OR (obesity)) | 336 |
| Web of science | **(((((((TS=(BFRT)) OR TS=(BFR)) OR TS=(blood flow restriction therapy)) OR TS=(blood flow restriction)) OR TS=(blood flow restriction training)) OR TS=(blood flow restriction exercise)) OR TS=(KAATSU training))**  **(((TS=(Overweight)) OR TS=(obese)) OR TS=(obesity))**  #1 AND #2 | 448 |
| Scopus | ( ( TITLE-ABS-KEY ( overweight ) OR TITLE-ABS-KEY ( obese ) OR TITLE-ABS-KEY ( obesity ) ) ) AND ( ( TITLE-ABS-KEY ( bfrt ) OR TITLE-ABS-KEY ( bfr ) OR TITLE-ABS-KEY ( blood AND flow AND restriction AND therapy ) OR TITLE-ABS-KEY ( blood AND flow AND restriction ) OR TITLE-ABS-KEY ( blood AND flow AND restriction AND training ) OR TITLE-ABS-KEY ( blood AND flow AND restriction AND exercise ) OR TITLE-ABS-KEY ( kaatsu AND training ) ) ) | 515 |
| Embase | bfrt OR bfr OR 'blood flow restriction therapy'/exp OR 'blood flow restriction therapy' OR (('blood'/exp OR blood) AND ('flow'/exp OR flow) AND ('restriction'/exp OR restriction) AND ('therapy'/exp OR therapy)) OR 'blood flow restriction'/exp OR 'blood flow restriction' OR (('blood'/exp OR blood) AND ('flow'/exp OR flow) AND ('restriction'/exp OR restriction)) OR 'blood flow restriction training'/exp OR 'blood flow restriction training' OR (('blood'/exp OR blood) AND ('flow'/exp OR flow) AND ('restriction'/exp OR restriction) AND ('training'/exp OR training)) OR 'blood flow restriction exercise'/exp OR 'blood flow restriction exercise' OR (('blood'/exp OR blood) AND ('flow'/exp OR flow) AND ('restriction'/exp OR restriction) AND ('exercise'/exp OR exercise)) OR 'kaatsu training' OR (kaatsu AND ('training'/exp OR training))  'overweight'/exp OR overweight OR obese OR 'obesity'/exp OR obesity  #1 AND #2 | 912 |
| Cochrane library | (blood flow restriction exercise):ti,ab,kw OR (KAATSU training)  (BFRT):ti,ab,kw OR (BFR):ti,ab,kw OR (blood flow restriction therapy):ti,ab,kw OR (blood flow restriction):ti,ab,kw OR (blood flow restriction training):ti,ab,kw  (Overweight):ti,ab,kw OR (obese):ti,ab,kw OR (obesity):ti,ab,kw  #1 OR #2 AND #3 | 153 |
